# Ultrasonographic Evaluation of Three Approaches for Botulinum Toxin Injection into Tibialis Posterior Muscle in Chronic Stroke Patients with Equinovarus Foot: An Observational Study

**DOI:** 10.3390/toxins13110829

**Published:** 2021-11-22

**Authors:** Stefania Spina, Salvatore Facciorusso, Chiara Botticelli, Domenico Intiso, Maurizio Ranieri, Antonio Colamaria, Pietro Fiore, Chiara Ciritella, François Genêt, Andrea Santamato

**Affiliations:** 1 Spasticity and Movement Disorders “ReSTaRt” Unit, Physical Medicine and Rehabilitation Section, Policlinico Riuniti Hospital, University of Foggia, 71122 Foggia, Italy; chiarabott@gmail.com (C.B.); chiara.ciritella@gmail.com (C.C.); 2 Villa Beretta Rehabilitation Center, Valduce Hospital, Costa Masnaga, 23845 Lecco, Italy; s.facciorusso89@gmail.com; 3 Unit of Neuro-Rehabilitation, and Rehabilitation Medicine, IRCCS “Casa Sollievo della Sofferenza”, San Giovanni Rotondo, 71013 Foggia, Italy; d.intiso@operapadrepio.it; 4 Department of Basic Sciences, Neuroscience and Sense Organs, Aldo Moro University, 70124 Bari, Italy; maurizio.ranieri@uniba.it; 5 Department of Neurosurgery, University of Foggia, 71122 Foggia, Italy; colamariaa@gmail.com; 6 Neurorehabilitation Unit, IRCCS Maugeri, 70124 Bari, Italy; pietro.fiore@unifg.it; 7 UPOH (Unité Péri Opératoire du Handicap, Perioperative Disability Unit), Physical Medicine and Rehabilitathion Department, Raymond-Poincaré Hospital, GHU APHP-Université PARIS SACLAY, 92380 Garches, France; francois.genet@aphp.fr

**Keywords:** BoNT-A ultrasound-guided injection, spastic equinovarus foot, tibialis posterior muscle, stroke

## Abstract

Spastic equinovarus (SEV) foot deformity is commonly observed in patients with post-stroke spasticity. Tibialis posterior (TP) is a common target for botulinum toxin type-A (BoNT-A) injection, as a first-line treatment in non-fixed SEV deformity. For this deep muscle, ultrasonographic guidance is crucial to achieving maximum accuracy for the BoNT-A injection. In current clinical practice, there are three approaches to target the TP: an anterior, a posteromedial, and a posterior. To date, previous studies have failed to identify the best approach for needle insertion into TP. To explore the ultrasonographic characteristics of these approaches, we investigated affected and unaffected legs of 25 stroke patients with SEV treated with BoNT-A. We evaluated the qualitative (echo intensity) and quantitative (muscle depth, muscle thickness, overlying muscle, subcutaneous tissue, cross-sectional area) ultrasound characteristics of the three approaches for TP injection. In our sample, we observed significant differences among almost all the parameters of the three approaches, except for the safety window. Moreover, our analysis showed significant differences in cross-sectional area between treated and untreated. Advantages and disadvantages of each approach were investigated. Our findings can thus provide a suitable reference for clinical settings, especially for novice operators.

## 1. Introduction

Post-stroke spasticity has a prevalence of 25.3% after stroke and an incidence of 39.5% in patients with paresis [1]. Spastic equinovarus (SEV) foot deformity is commonly observed in patients with post-stroke spasticity and cerebral palsy. It is one of the seven postures and common patterns of lower-limb spasticity and it is characterized by ankle plantarflexion and inversion [2]. This deformity often causes significant problems with dressing and in particular shoe wearing, standing, transfer, and walking. It also has an impact on pain, activities of daily life, caregiver burden and the quality of life [3,4,5].

Spastic equinovarus foot has four main causes: (1) spasticity of the calf muscle, (2) shortening of the spastic muscle, (3) weakness of the ankle dorsiflexor muscle, and (4) imbalance between the tibialis anterior and the peroneus muscle [6]. To address SEV deformity, a constellation of procedures may be used, including physiotherapy, muscle stretch training, use of orthosis, surgery such as tendon transfer, tendon lengthening, and bone surgery, alcohol phenol neurolysis, selective neurotomy, or botulinum toxin type-A (BoNT-A) injection. The therapeutic decision varies from patient to patient, and it is not infrequent that different procedures should be combined. Among all the options, BoNT-A is considered the first-line treatment of focal spasticity with a high level of evidence and it has been established as safe and effective [7]. Several studies have established its effect on reducing ankle plantar flexor spasticity [8], pain [9], improving walking [10,11]. Finally, a recent meta-analysis has demonstrated the efficacy of BoNT-A in lower extremity spasticity following stroke, improving both muscle tone and functional outcomes [12].

Muscles that potentially contribute to equinovarus foot deformity include the tibialis posterior, medial and lateral gastrocnemius, soleus, the long toe flexors, and extensor hallucis longus. Among these, the tibialis posterior is considered a possible contributor to the varus deformity as it is the major foot invertor. In almost all the cases, experts agreed to target the TP for BoNT-A injection in SEV deformity [2,13]. Needle insertion in the tibialis posterior is critical due to its localization in the deep division of the posterior compartment of the lower leg and its closeness to neurovascular bundles. Therefore, for this deep muscle an instrumental guide, such as electromyography, electrical stimulation or ultrasonography (US) is strongly recommended to guarantee administration accuracy and thus improve the clinical outcome [14,15]. We choose the US guidance as it is widely available, time saving, cost-effective and pain free and allows us to visualize the muscle and characterize its alteration due to immobilization and spasticity [16].

In current practice, there are several ways to inject the tibialis posterior muscle: an anterior (AA), posteromedial (MA), or posterior approach (PA). The ultrasonography anterior approach was described by Rha [17] in healthy volunteers. In this approach the patient was supine, and the probe was placed over the anterior tibial: the upper third was found to be an adequate place for needle placement due to a larger safety window even in the presence of deeper distance. Subsequently, the same author compared the posterior and anterior ultrasonographic approach in hemiparetic children with cerebral palsy suggesting needle placement at the upper third point of the tibia for the anterior approach and at the midpoint for the posterior approach [18]. The same result was confirmed by Won [19] who had assessed in healthy volunteers four different points for needle insertion comparing anterior and posterior approaches; he concluded that the most favorable is the posterior approach at the midpoint of the length between the tibial tubercle to the bimalleolar line followed by the anterior approach at the upper third of the tibia. Finally, the medial approach was explored by Picelli et al. [20] giving information about the muscle depth and thickness in hemiparetic stroke patients; in this study the tibialis posterior was imaged by placing the probe at about 50% of a line between the medial femoral condyle to the medial malleolus. Other cadaveric [21] or lower extremity magnetic resonance imaging studies exploring the safest approach to needle electrode insertion into TP, found that the anterior approach could offer a larger safety window width at the upper third of the tibia compared to the posterior approach.

To date, there is no established technique for the location of the needle insertion into the tibialis posterior muscle and a much-debated question is whether ultrasound window is the safest and suitable for spastic patients.

The aim of this study was to examine and compare the ultrasonographic characteristic of the three approaches for the TP location and injection in chronic stroke patients with equinovarus foot. Secondly, the study set out to evaluate the muscle alteration due to spasticity and BoNT-A injection comparing the affected and the unaffected lower limb.

## 2. Results

We enrolled 25 consecutive chronic stroke patients. All subjects previously received BoNT-A injection for the equinovarus foot and in all cases the TP was infiltrated. Table 1 shows the demographic data and clinical feature of our sample.

The Wilcoxon Test was used to compare unaffected and affected sides showed a significant difference in muscle thickness in the anterior and posterior approaches and in cross sectional area (CSA). All the other calculated parameters did not show statistically significant differences, as shown in Table 2.

A Spearman rho correlation was investigated among characteristics and parameters evaluated. Subcutaneous tissue in every approach shows a statistically significant correlation with gender (AA: *rho* = 0.506; *p* < 0.01; MA: *rho* = 0.579; *p* < 0.002; PA: *rho* = 0.550; *p* < 0.004). In males, the subcutaneous tissue is thinner. Muscle depth significantly correlated with gender in medial and posterior. There is a strong relationship between cross-sectional area and posterior muscle thickness in the affected (*rho*: 0.838; *p* < 0.001) and unaffected side (*rho*: 0.759; *p* < 0.001). CSA positively correlated with FAC score (*rho*: 0.455; *p* < 0.022) and age (*rho*: −0.554; *p* < 0.004): the greater the CSA of TP, the higher the FAC score. The higher the age, the lower the CSA of TP. A moderate correlation was found between overlying muscle and muscle depth in PA (*rho*: 0.607; *p* < 0.001) and AA (*rho*: 0.660; *p* < 0.001). No other correlations were found.

A Friedman test and pairwise comparisons with Bonferroni’s correction were performed to compare the average measures in the affected side for AA, MA, and PA (Table 3). A significant difference was found in depth, thickness, and subcutaneous tissue. No significant difference was found for the safety window.

## 3. Discussion

This is to our knowledge the first study to describe and compare the three ultrasonographic approaches to tibialis posterior muscle for the BoNT-A injection in spastic equinovarus foot post-stroke.

Very little was found in the literature on the question of which approach is better for having a good ultrasonographic image of TP and there is no confirmed technique for the location of the needle insertion into tibialis posterior for BoNT-A injection. It is now well established that determining the target injection site is the most important procedure in the treatment with BoNT-A: it is crucial to be as precise as possible in order to guarantee the best efficacy in the target muscle and to avoid any unwanted effects due to toxin diffusion or injury of neurovascular bundles [14,22]. Of note, some blind or landmark guided injections would no longer be considered to be reasonable alternatives in this era of technology. In fact, manual placement of the needle in the muscle has been shown to be inaccurate especially for deep muscle [23]. Moreover, it has been seen that the accuracy of manual placement in the tibialis posterior was only 11% compared to electrical stimulation [24]. It is clear that a guide should be used; the clinicians have different options such as electrical stimulation, electromyography and ultrasonography (US). At the present, no instrument-based guiding technique was proved superior to another and no recommendation can be made in terms of choosing the optimal guiding technique [25]. In this study, we choose the ultrasound as an imaging assistance guide because it allows direct or indirect visualization of the needle, the muscle target, and the neurovascular bundle to be avoided and in addition, it is painless and fast. However, ultrasound quality is operator-dependent and subjective to interpretive error: to provide the appropriate BoNT-A treatment the practitioners should be well informed of the anatomy of the target area and must be confident with the ultrasound technique.

The TP muscle takes its origin from the posterior surface of the upper half of the posterior tibia, the middle of the posterior fibula, and the posterior interosseous membrane. The muscle belly runs posterior to the interosseous membrane, between the flexor digitorum longus (medial) and the flexor hallucis longus (lateral) muscles which usually originate below the TP at the middle third of the leg. Along its entire course, the tibialis posterior muscle has a close relationship with the posterior neurovascular bundle of the leg (formed by the posterior tibial artery and veins and the tibial nerve) that lies between it (anterior) and the soleus muscle (posterior).

The current study found that the three ultrasonographic approaches to TP have significant differences in almost all the parameters evaluated. Considering the affected leg, the major depth of TP was found in the posterior approach (30.64 ± 3.46 mm) followed by the anterior (26.96 ± 3.46 mm). In the posteromedial approach, the TP has the lowest depth (22.50 ± 2.15 mm) and the major thickness due to the scan of its major axis (its oval shape). The subcutaneous tissue is similar in the posteromedial and posterior approach and less in the anterior approach, while the overlying muscle is major in anterior and posterior approach compared to the medial. The safety window is smaller in the medial approach and bigger in the anterior approach without a statistically significant difference.

We have detected a significant relationship between gender and subcutaneous tissue in both posterior approaches (MA and PA). Indeed, the subcutaneous tissue in these approaches is significantly greater than the anterior approach. In females, the anterior approach can be a more convenient choice to inject when subcutaneous tissue is thicker. The relationship found between the overlying muscle and depth could be another relevant data for a better choice of injection window. Our analysis showed a thicker overlying muscle in the anterior (AA) and posterior approach (PA). The choice of the medial approach can be better in the case of patients with thicker overlying muscles.

It was not determined which of the three approaches is better in clinical practice because each approach has advantages and disadvantages and other factors rather than only the anatomic parameters should be taken into account. One of these factors is the location of the motor entry point (MEP) defined as the location where motor nerve penetrated the muscle belly, and intramuscular motor point (IMP) defined where the intramuscular motor nerve ends. There is a lack of consensus in the literature about the superiority of targeted injections, although results from studies indicate that targeting MEP and IMP zones can increase the effectiveness of BoNT-A injections [26,27,28]. The existing literature has not yet determined the optimal anatomical site for an effective botulinum toxin injection in TP. Lee et al. [29] elucidated the anatomical location of the MEP and IMP of the TP respectively as a dense area in the 10–30% region, and as dense area in the 10–40% and 70–80% regions of a line between the proximal medial articular margin of the tibia and the most distal point the malleolus of the tibia. Some years later Yi et al. [30] observed an intramuscular nerve arborization pattern into tibialis posterior at 70–80% of a line between the lateral malleolus (0%) and the fibular head (100%). These areas can be used for the injection; the clinician could choose two different approaches to target at least two different zones following the guidelines suggesting that the sites injection in TP should be 1 to 3.

Regarding the injection technique, the clinician should take into account some important points: a comfortable position for both patient and clinician should be obtained [31], the injection site should be fully exposed, the probe should be perpendicular to the plane of the target limb and the needle could be inserted along the longitudinal axis (in-plane) or short axis (out of plane) of the probe. In our practice, we usually performed an out of plane injection into TP that allowed us a more comfortable needle entry angle and less distance to the target.

**Figure 1:**
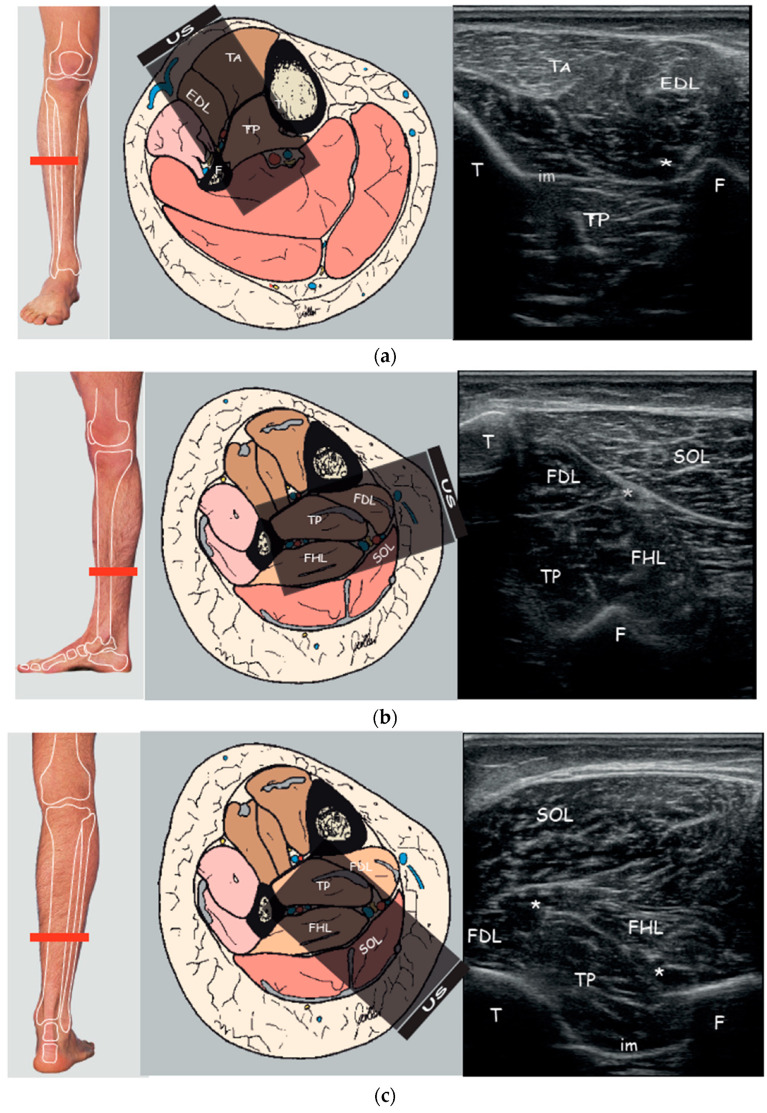
Right to left: Probe position to evaluate tibialis posterior on the axial plane; Anatomical scheme of axial section of the leg correlated with US scan; representative US axial real scan, healthy subject. (**a**) Anterior approach; (**b**) Posteromedial approach; (**c**) Posterior approach. Abbreviations: *TA* tibialis anterior muscle; *EDL* extensor digitorum longus muscle; *TP* tibialis posterior muscle; *SOL* soleus muscle; *FDL* flexor digitorum longus muscle; *FHL* flexor hallucis longus muscle; *T* tibia; *F* fibula; *im* interosseous membrane; * neurovascular bundle.

During the anterior approach (Figure 1a), the TP is reached at the upper third of the leg after passing the tibialis anterior and the interosseous membrane which appears as a hyperechoic band between the tibia and the fibula; passing the membrane could lead to more pain to the patient. Moreover, attention must be paid to the anterior neurovascular bundle (anterior tibial artery and vein and deep peroneal nerve) running close to the posterolateral border of the tibia. Although it is indeed easy to place the probe and to access the TP by this view, we usually do not perform this technique due to lack of visibility of the whole belly muscle behind the tibia, the prerequisite to use a needle more than 30 mm long, the need to perforate the interosseous membrane and the resultant increased pain for the patient. Moreover, a recent study characterizing the microscopic structure and sensory nerve endings of the interosseus membrane had found that interosseous membrane may play a role in proprioception [32]. Therefore, it is better to preserve such structure from being perforated by the needle and save any possible mechanoreceptors as possible.

During the posteromedial approach (Figure 1b), the patient lies supine with the leg extra rotated; the probe is placed in the distal third of the leg and the needle should be passed through a thin layer of soleus and flexor digitorum longus muscle (FDL) between the posterolateral border of the tibia and the posterior tibial neurovascular bundle. The advantages of this technique are the minor depth of the muscle that can be reached using the 30 mm needle, the less overlying muscle layer and so the minor muscle tissue to pass, the major thickness of TP due to the scan over its major axis and the less risk to overpassing the belly muscle.

During the posterior approach (Figure 1c), the TP is reached after passing the soleus and the flexor hallucis longus and between the posterior neurovascular bundle (posterior tibial artery and vein and tibial nerve) medially and the peroneal artery and vein laterally. We should not advise this access for novice operators because it is more difficult to distinguish the border of the TP and to differentiate the TP from other structures; it could be asked of the patient to actively move his toes or the clinician could passively move them in order to visualize better the adjacent muscle (FHL, FLD) thus isolating the TP. Moreover, due to the depth of TP (30.64 ± 3.46 mm) it cannot be used the routine needle length of 30 mm. Finally, the prone position of the patient may be difficult to reach or could be uncomfortable for a patient with hemiparesis post-stroke.

Nevertheless, the posterior approach is the only scan that could guarantee the visualization of the whole muscle belly of the TP and it is crucial for the cross-sectional area (CSA) measurement. In fact, in any transverse view of ultrasonography by the anterior or by the medial approach it was possible to show the whole cross-sectional image of the TP. TP could potentially be obscured by either the tibia or fibula and the overlying structure such as the interosseous membrane or fibrous muscles could alter the view. Therefore, in contrast to Johnson et al. [33] who recommend measuring the CSA of TP using the anterior view at 30% of the shank length, we noted that it was difficult to image and measure the whole CSA at this level in our patients. We then measured the CSA by a posterior approach at the junction of middle and lower limb: at this level, the image was clearer as it was possible to visualize all the TP border and the measurement depth is reduced compared to the proximal leg because it is necessary to scan through only the soleus and not the gastrocnemius. We saw a correlation between CSA and age: the older the patient, the smaller the muscle size. Age is considered an independent risk factor for sarcopenia and muscle mass progressively decreases as humans get older [34]. Moreover, CSA is correlated with ambulation ability. The significant relationship between FAC and CSA highlights how the TP in the affected leg can play a functional role in the gait of the patient. In this case, posteromedial approach (MA) could be the right choice as we showed a greater thickness in this window. This made sense since we demonstrated that muscle thickness was related to the CSA in posterior approach.

In this study, we compared the TP cross-sectional area of the affected side (31.42 ± 3.66 mm) to the contralateral unaffected side (36.09 ± 5.27 mm) to determine the effects of stroke and BoNT-A injections on muscle. It was found a statistically significant difference and this is not surprising because it is known that spasticity may affect the normal muscle architecture leading to increased variability of the size and type of muscle fibres, decreased numbers of sarcomeres, a proliferation of the extracellular matrix with an increased collagen concentration [35,36]. Moreover, BoNT-A injections may have an additional impact on muscle structure leading to atrophy, remodeling of contractile proteins, and change in muscle elasticity [37]. However, it is beyond the scope of this study to differentiate the consequences related to spastic paresis from the ones related to injections of botulinum toxin.

Moreover, the aging and the disuse due to reduced physical activity and compensatory motor patterns could lead to muscle atrophy and weakness [38]. In our sample, one patient performed only therapeutic walking, 10 patients were indoor ambulators 14 patients were outdoor ambulators. The importance of activity level is proved by the fact that both affected and unaffected limbs adapt following stroke when compared to age-matched muscle. Therefore, post-stroke muscular alterations should be considered a multi-factorial phenomenon.

We further observed higher echo intensity measured with the Modified Heckmatt scale [39] in the affected leg compared to the unaffected leg. The increased echo intensity suggests that spastic paresis could lead to fatty infiltration or fibrosis in the muscle affected. Moreover, spastic muscles with higher echo intensity may have a reduced response to BoNT-A [40]. Ultrasound allows observing these muscle alterations and thus could help to choose the best site for BoNT-A administration.

Finally, in Table 4 we summarize the possible advantages and disadvantage of the three approaches.

Several limitations to this study need to be acknowledged. First, the sample size is small, and it is limited to adult patients with spastic equinovarus foot due to stroke. Accordingly, a study in a large cohort is warranted. Moreover, it would be interesting to assess the ultrasound characteristics of TP in adult and non-adult patients with PES due to different etiologies. Second, we did not evaluate and compare the accuracy and the effects of BoNT-A injection in TP using the three different ultrasonographic approaches. Future research should focus on determining the superiority of a BoNT-A injection approach in TP on the functional outcome. Third, another source of uncertainty has been the possibility of measurement error on the ultrasound image; for this reason, it would be beneficial to compare TP’s measurements via ultrasound and via MR scan in the same individual. Fourth, in our sample, it was not possible to differentiate in the affected side the effect on muscle structure of paresis or BoNT-A injections. It would be useful to include another group of patients with SEV due to stroke non-treated with BoNT-A. Fifth, all our patients were assessed lying on the examination bed either in supine or prone position. In clinical practice, some patients cannot be transferred to the examining bed and needed to be injected while sitting in their wheelchair. Injector could choose an anterior or posteromedial approach. In this position, the muscle features should not be much different from the supine position, but the reader should be aware that our data refer to the lying position.

## 4. Conclusions

To our knowledge these are the first data that investigated, the three approaches for TP BoNT-A injection by means of ultrasonography and compared the TP characteristics of the affected and unaffected sides.

Our findings show that there are significant differences among most of the parameters evaluated. Considering the presence of neurovascular bundle, the little CSA and depth of TP, we can speculate that ultrasound should be considered to optimize clinical outcomes for BoNT-A treatment. The differences observed between affected (injected), and unaffected (uninjected) sides could be due to effects of BoNT-A injection on the rheological properties.

The observations by these data can thus provide a suitable reference for clinical settings, especially for novice operators. Further studies are needed to assess differences in clinical outcomes of these three approaches and compare rheological characteristics in chronic stroke patients with SEV naive to BoNT-A treatment.

## 5. Materials and Methods

An observational cohort study was performed from December 2020 to April 2021.

Twenty-five chronic stroke patients (M = 13; F = 12) were enrolled in this study. All participants (age > 18) were outpatients treated with BoNT-A with last injection > 3 months; Other inclusion criteria were: time since event > 6 months, spastic equinovarus foot with >2 at Modified Ashworth Scale [41] (MAS). We excluded patients with fixed contractures or bony deformities; previous surgical or neurolytic procedures of affected lower limb; intrathecal pharmacological treatment (i.e., baclofen). All participants did not have adjunctive therapies post-injection. Written informed consent for participation in the study was carried out according to the Declaration of Helsinki and validated by the local Ethics Committee and Competent Authority.

All patients underwent real-time B-mode ultrasonography using a MyLab™ Seven system (Esaote SpA, Genoa, Italy) equipped with a 3–13 Hz linear-array transducer “SL1543”. The settings were the same for all patients. A musculoskeletal preset was used (Tissue Enhanced Imaging mode; XView Algorithm: +5; MView algorithm: 2; Persistence: 0; Dynamic range/Dynamic compression/Density/Gray map: 11/1/1/0; Mechanical Index 1.3); depth was set at 52 mm, focus zone was placed manually based on the tibialis posterior depth. The probe was placed over the skin perpendicular to the tibial-fibular axis in a long-axis view. Minimal pressure was applied until the whole probe line was in contact with the. A water-soluble transmission gel was used between the transducer and the skin to displace air from the transducer-skin interface.

The TP muscle was identified on the surface using EUROMUSCULUS/USPRM spasticity approach for the anterior approach (25% of the distance from the fibular head to the lateral malleolus, behind the posterior border of the tibia) [42]; for the posteromedial approach, the TP muscle was located on the posteromedial surface of the leg using the Silvestri Muda Orlandi (distal third of the leg) [43]; the posterior approach was evaluated on the posterior surface at the at the junction of the middle and lower thirds of the leg with the probe placed at the posterior side of the leg perpendicular to the virtual line extending from the middle of the popliteal fossa to the intermalleolar line [44] (Figure 1).

The same physician with at least 3 years of experience in BoNT-A ultrasound-guided injection acquired and stored three transverse ultrasonographic scans. The mean of the three measures was calculated for statistical analysis. The transducer was removed from the leg between trials. The images were processed with ImageJ (National Institutes of Health, Bethesda, MD, USA) to measure muscle depth (MD), subcutaneous tissue (ST) and overlying muscle (OM), safety window (SW) and muscle thickness (MT), and echo intensity in affected and unaffected side with Modified Heckmatt scale (grade 1 = normal echogenicity in greater than 90% of the muscle; grade 2 = increased muscle echogenicity in 10–50% of tissue; grade 3 = marked increase in muscle echogenicity between 50–90% of tissue; grade 4 = very strong muscle echogenicity in >90% of tissue) [39] evaluated on the anterior approach in all subjects.

During the processing phase of images stored, first it was identified the TP by an experienced physician and then all the parameters were evaluated. The MT was measured as the distance between the superficial and the deep fascia of the TP at the widest distance; the MD as the distance between the upper aponeurosis of the TP and the line separating the dermis from fat; the ST as the distance between the line separating the derma from fat and the upper aponeurosis of the first muscle layer above the TP; the OM as the distance between the superficial and the deep aponeurosis of the first muscle layer above the TP that with some variability between subjects, is composed of tibialis anterior in the AA, flexor digitorum longus and soleus in MA and soleus and flexor hallucis longus in PA.

We evaluated MT and MD that were measured as the distance between superficial and deep aponeuroses and between superficial aponeurosis and the line separating the dermis from fat, respectively.

**Figure 2:**
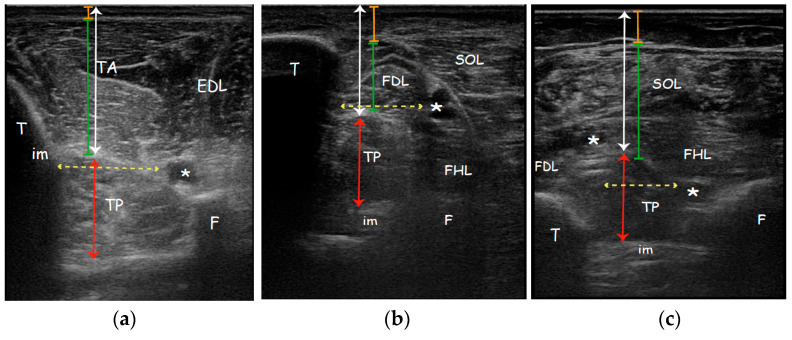
Real ultrasound images of a patient enrolled in the study, affected side. Parameters measured with ultrasonography evaluating the (**a**) Anterior approach; (**b**) Medial approach; (**c**) Posterior approach. Orange line: subcutaneous tissue thickness; Green line: overlying muscle thickness; White arrow: TP muscle depth; Red arrow: TP muscle thickness; Yellow dotted arrow: safety window. Abbreviations: TA tibialis anterior muscle; EDL extensor digitorum longus muscle; TP tibialis posterior muscle; SOL soleus muscle; FDL flexor digitorum longus muscle; FHL flexor hallucis longus muscle; T tibia; F fibula; im interosseous membrane; * neurovascular bundle.

Cross-sectional area (CSA) was measured only in the posterior approach since only this view ensures a complete visualization of the TP and it was represented by the total area of the TP muscle surrounded by the muscle fascia in a transverse scan perpendicular to the direction of muscle fibres [45]. Safety window (SW), defined as the distance between structure that must be avoided during needle insertion to prevent a neurovascular damage, in AA was calculated between tibialis anterior artery (adjacent to the branches of fibular nerve) and medial side of the tibia. SW in MA was measured between tibialis posterior artery and posterior side of the tibia. SW in PA was found between tibialis posterior artery and fibular artery (adjacent to the fibula and deep to the flexor hallucis longus) (Figure 2). Safety window was calculated only on the affected side.

During evaluation of the anterior approach, subjects were placed in the supine position while the posterior approach was taken with patients in prone position. To avoid inter-individual variability, all measurements were taken by the same clinician.

As clinical outcome measures were used Modified Ashworth scale (MAS) to evaluate plantar-flexors spasticity, Functional Ambulation Classification (FAC) [46] and Walking Handicap Scale [47] to evaluate ambulation ability.

We performed a descriptive statistic to analyze all variables. Quantitative variables were reported as mean ± standard deviation (SD). Ordinal variables were reported with median. Normality of distribution was checked by the Shapiro–Wilk’s test. The difference among three approaches on the affected side were analyzed with nonparametric Friedman test and a pairwise comparison with Bonferroni correction. The differences between affected and unaffected hemiparetic side were analyzed through a nonparametric Wilcoxon sample test. A Spearman rho correlation test was performed to investigate the relationship between sonographic measures and Modified Heckmatt scale (MHS), clinical outcome, and demographic characteristics. Statistical analyses were conducted using Statistical Package for social science (IBM SPSS Inc., Armonk, NY, USA). The level of significance was set to *p* < 0.05.

## Figures and Tables

**Table 1:** Demographic and clinical features of all patients (*n* = 25).

**Age (years)** *mean* ± *SD*	59.25 ± 11.28
**Gender** *(Male/Female)*	13/12
**Time since event (years)** *mean* ± *SD*	5.58 ± 5.68
**Side affected** *(right/left)*	16/9
**Type of stroke** *(Ischemic/Haemorrhagic)*	15/10
**BMI (kg/m^2^)** *mean* ± *SD*	26.67 ± 3.58
**MAS** *Median (min–max)*	2 (2–3)
**Echogenicity in affected side Modified Heckmatt scale** *Median (min–max)*	3 (2–3)
**Echogenicity in unaffected side Modified Heckmatt scale** *Median (min–max)*	2 (2–3)
**FAC** *Median (min–max)*	4 (1–4)
**WHS** *Median (min–max)*	4 (2–5)

Abbreviations: SD, Standard Deviation; BMI, Body Mass Index; MAS, Modified Ashworth Scale; FAC, Functional Ambulation Classification; WHS, Walking Handicap Scale.

**Table 2:** Comparison between affected and unaffected side.

		Affected Side Mean ± *SD*	Unaffected Side Mean ± *SD*	*p*-Value
Anterior approach	TP muscle depth (mm)	26.96 ± 3.07	27.29 ± 2.63	0.647
	Subcutaneous thickness (mm)	4.62 ± 2.37	4.86 ± 1.44	0.090
	Overlying muscle thickness (mm)	22.34 ± 3.31	22.42 ± 2.64	0.904
	TP muscle thickness (mm)	14.66 ± 1.34	15.62 ± 1.20	0.007 *
	Safety window (mm)	14.39 ± 2.36	-	-
Medial approach	TP muscle depth (mm)	22.50 ± 3.69	21.68 ± 3.74	0.317
	Subcutaneous thickness (mm)	7.93 ± 3.72	7.68 ± 2.59	0.798
	Overlying muscle thickness (mm)	14.57 ± 1.81	13.99 ± 2.92	0.412
	TP muscle thickness (mm)	21.87 ± 1.74	22.32 ± 1.75	0.300
	Safety window (mm)	12.72 ± 2.50	-	-
Posterior approach	TP muscle depth (mm)	29.76± 3.52	29.12 ± 2.45	0.353
	Subcutaneous thickness (mm)	7.74 ± 2.67	7.34 ± 2.43	0.467
	Overlying muscle thickness (mm)	22.42 ± 2.64	21.78 ± 1.66	0.139
	TP muscle thickness (mm)	15.07 ± 0.55	15.69 ± 1.05	0.015 *
	Safety window (mm)	11.97 ± 0.95	-	-
	Cross-sectional area (mm^2^)	31.42 ± 3.66	36.09 ± 5.27	<0.001 *

Abbreviations: TP, tibialis posterior. * Significance level *p* < 0.05.

**Table 3:** Friedman test and post hoc analysis for three approaches of tibialis posterior.

				Anterior—Medial	Anterior—Posterior	Medial—Posterior
	Χ^2^	df	*p-*Value	*p*-Value *		
Depth	36.273	2	*p* < 0.001	0.004	0.033	<0.001
Thickness	39.120	2	*p* < 0.001	<0.001	0.609	<0.001
Subcutaneous tissue	26.727	2	*p* < 0.001	<0.001	<0.001	1.000
Overlying muscle	35.280	2	*p* < 0.001	<0.001	1.000	<0.001
Safety window	5.840	2	*p* > 0.05	-	-	-

Abbreviations: df, degrees of freedom * Significance *p*-values have been adjusted by the Bonferroni correction for multiple tests. Significance level *p* < 0.05 indicated by italics.

**Table 4:** List of the possible advantages and disadvantages of the three approaches.

	Anterior Approach	Medial Approach	Posterior Approach
Advantages	Patient supine position Less subcutaneous tissue Easy US TP recognition Proximity to MEP	Patient supine position Less TP depth Less overlying muscle More TP thickness	Full visualization of target
Disadvantage	Partial display of target More TP depth More overlying muscle IM perforation Painful infiltrative technique	Partial display of target Distance to MEP	Patient prone position Difficult US TP recognition More TP depth More subcutaneous tissue More overlying muscle Distance to MEP

Abbreviations: TP: tibialis posterior; US: ultrasound; MEP: motor end plate; IM: interosseus membrane.

## Data Availability

Not applicable.
